# An accuracy measurement method for star trackers based on direct astronomic observation

**DOI:** 10.1038/srep22593

**Published:** 2016-03-07

**Authors:** Ting Sun, Fei Xing, Xiaochu Wang, Zheng You, Daping Chu

**Affiliations:** 1 Department of Precision Instrument, Tsinghua University, Beijing, China; 2 Electrical Engineering Division, Department of Engineering, University of Cambridge, United Kingdom; 3 State Key Laboratory of Precision Measurement Technology and Instruments, Tsinghua University, Beijing, China; 4 Qian Xuesen Laboratory of Space Technology, China Academy of Space Technology, Beijing, China

## Abstract

Star tracker is one of the most promising optical attitude measurement devices and it is widely used in spacecraft for its high accuracy. However, how to realize and verify such an accuracy remains a crucial but unsolved issue until now. The authenticity of the accuracy measurement method of a star tracker will eventually determine the satellite performance. A new and robust accuracy measurement method for a star tracker based on the direct astronomical observation is proposed here. In comparison with the conventional method with simulated stars, this method utilizes real navigation stars as observation targets which makes the measurement results more authoritative and authentic. Transformations between different coordinate systems are conducted on the account of the precision movements of the Earth, and the error curves of directional vectors are obtained along the three axes. Based on error analysis and accuracy definitions, a three-axis accuracy evaluation criterion has been proposed in this paper, which could determine pointing and rolling accuracy of a star tracker directly. Experimental measurements confirm that this method is effective and convenient to implement. Such a measurement environment is close to the in-orbit conditions and it can satisfy the stringent requirement for high-accuracy star trackers.

With the development of earth-observation, deep-space exploration and celestial navigation, the requirements for attitude measurement are rapidly increasing. Its accuracy determines the spacecraft performance and it is a crucial factor to the completion of a space mission. Star tracker is an important and promising attitude measurement device with the highest accuracy among different types of attitude measurement devices^1^,^2^,^3^,^4^,^5^,^6^. It is the main source of the attitude information for a spacecraft. The GeoEye-1 satellite^7^ can acquire images with high resolution (0.4 m) and high geo-location (5 m @ CE90)^8^, mainly due to the High Accuracy Star Tracker (HAST) developed by Ball Corp.^9^. The Pléiades-1B^10^ can provide high resolution (0.5 m) and location (5.3 m @ CE90) images. It employs three dedicated star tracker SED36 developed by Sodern Corp. HYDRA^11^ is a new generation star tracker developed by Sodern Corp. and applied to SPOT-6 satellite and it consists of three heads. In addition, ESA Darwin mission is a concept designed to directly detect Earth-like planets orbiting around the nearby stars and search for evidence of life on these planets. It also considers using high accuracy star tracker as its key control component^12^. Since the star tracker is much needed for high-accuracy attitude determination, it is essential that its high accuracy can be realized and verified. However, the real accuracy is difficult to measure completely in a laboratory or in in-orbit conditions. Therefore, it is desirable to develop a suitable accuracy measurement method, which is easy to implement and perform and can meet the high accuracy requirements.

In 2001 and 2002, Liebe^13^ and Ju^14^ introduced the use of single star accuracy as a reference to evaluate the star tracker performance. Obviously, it only represents the attitude accuracy to some extent. Since then different laboratory calibration and test methods have been widely discussed. Xing *et al.* presented several effective laboratory calibration methods^15^,^16^,^17^,^18^,^19^,^20^,^21^ with different calibration models and procedures. Camera calibration methods^22^,^23^,^24^,^25^,^26^ based on computer vision techniques are also used for the star tracker calibration since they have similar optical imaging principles and systems. With these methods, a range of parameters, such as the principal point, focal length, distortion and installation error, can be well calibrated. As a result, a successful calibration is also considered as an important reference that the accuracy satisfies the requirement. However, such laboratory based measurements or calibration methods have several disadvantages. Firstly, since the measurement is based on auxiliary facilities like the turntable and star simulator, the relationship between the star tracker and auxiliary facilities becomes complicated because of their coupling factors, which hinders the ability to achieve real and optimal results. Secondly, the actual laboratory environment is usually quite different from the real night sky which makes the accuracy result unreliable. In addition, the operation has many stingent requirements with regard to auxiliary facilities, such as an expensive precision turntable and a high-accuracy star simulator. Therefore, laboratory methods are more suitable for the normal calibration and single star accuracy evaluation. When more advance functions, such as star extraction, star recognition and attitude solution, are needed, an additional high performance star simulator will be required. In terms of the star simulator development, Hughes Aircraft Company^27^,^28^, McDonnell Douglas Aerospace^29^ and European Aeronautic Defense and Space Company^30^ have developed some advanced star simulators with superior star image simulation and stability. However, difficulties still exist at present in developing full celestial sphere star simulators which can satisfy the requirements for spectral range, magnitude, update rate and position accuracy simultaneously.

Another accuracy measurement method for star trackers is based on night sky observations. It utilizes telescopes or astronomical calculations. Real night sky experiment is an effective approach to evaluate and test the accuracy of star trackers with more accurate and realistic features. One of the approaches is to combine a star tracker with a telescope for high accuracy. Jørgensen^31^ and Denver^32^ conducted an accuracy measurement test that utilized real sky observations and telescopes and significantly improved the authenticity though the facilities involved were quite complex. Considering that the experiment needs to be performed with a high performance telescope, the measurement becomes highly dependent on the accuracy of the actual telescope in use. The operation is complicated and the measurement error is difficult to separate, which makes such a method not conducive to applications in various fields. The other approach is direct astronomic observation on ground as mentioned in^33^, whereas the measurement theory and evaluation system for accuracy measurement need additional researches.

Liu^34^, Schmidt^35^ and Lai^36^,^37^ proposed on-orbit measurement methods based on astronomy. Liu’s method mainly researches on on-orbit parameters calibration after launching. Schmidt’s method was designed for geo-stationary satellites. Lai’s method utilized the relationship between two star trackers on the same satellite platform. They are ingenious measurement methods under special conditions, while the methods are not suitable for the general development in particular in the early stage of measurements. Moreover, ground-based accuracy expression of the star tracker was not discussed.

We would like to develop a comprehensive method for star trackers which can take into consideration the motion of the Earth and the stars when conducting accuracy measurements based on real night sky observations. This method should have the merits of high accuracy, easy operation, less required auxiliary equipment and being close to the real conditions encountered in the orbit. In this paper, we propose an accuracy measurement method based on the inverse transformation of the Earth’s movement under real night sky and describes an accuracy expression approach. This method can represent the accuracy of three axes and it has the potential to become a measurement standard in future. It can also verify the functions of star pattern recognition, dynamic performance, star sensitivity, reliability and other technical specifications of a star tracker.

## Introduction

### Principle of the accuracy measurement method of a star tracker

#### Principle of the proposed accuracy measurement method

The Accuracy Measurement method of a Star Tracker based on direct Astronomic observation (AMSTA) takes the precise motion of the Earth as the reference for measurements. In use, the star tracker is fixed on the ground and moves along with the Earth. The proposed AMSTA method utilizes the Earth’s precise rotation. The optical axis is pointing near to the zenith to reduce atmospheric refraction effects as shown in Supplementary Fig. S1. The star tracker has the same motion including rotation around the Earth axis, precession around ecliptic axis, and nutation. After obtaining the exact motion of the Earth, the real-time inverse transformation of the attitude matrix or inverse transformation of the navigation star vector (see Supplementary Method) can be performed. After transformation, the pointing vector of the star tracker remains unchanged, and the corresponding measurement result is a kind of expression of the pointing and rolling accuracy of the star tracker. Therefore, it needs to make rigorous analysis and calculations regarding the motion of the Earth in the inertial space.

#### Motion of the Earth

**Figure 1:**
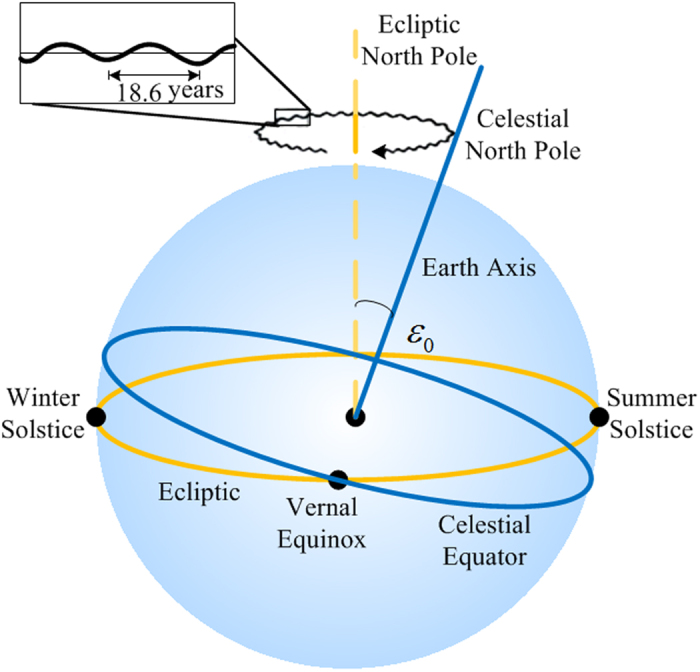
Main parameters of the Earth’s motion.

Figure 1 shows the celestial coordinate system and main parameters. An imaginary large sphere having any given radius and using the Earth as the center thereof is referred to as the “celestial sphere”. The Earth axis precesses slowly around an ecliptic pole in a given period, and an intersection line of the equatorial plane and an ecliptic plane may also rotate in the ecliptic plane in the same period. As shown in Fig. 1, a celestial north pole revolves around an ecliptic north pole clockwise with a radius of *ε*_0_ = 84381.448″. Given that the direction of the Earth’s revolution is opposite to the precession direction of the Earth axis, a small west movement of the vernal equinox is generated every year. This movement is referred to as a precession. Modern astronomical measurements and calculation results show that the precession of the Earth every year is Δ*ψ* = 50.290″. In this way, the celestial north pole revolves around the ecliptic north pole once about every 25 765 years^38^.

Similar to a gyro movement model, the nutation of the Earth axis exists in addition to the precession of the Earth axis. The nutation appears for complicated reasons but in general it is caused by the gravitation of other planets and the Moon to the Earth. Modern astronomical measurement results show that the period of the nutation is 18.6 years (6798 days), a nutation component in longitude on the ecliptic is 17.24″ and an oblique nutation component perpendicular to the ecliptic is 9.21″^39^. Therefore, the coordinates such as the right ascension and the declination will change accordingly.

Polar motion of the rotation axis of the Earth also exists, however, the periodic change of the polar motion is below 0.1″, which can be disregarded considering the accuracy of the star tracker.

In summary, the movement of the Earth in the inertial space includes rotation about its axis, precession with respect to the ecliptic north pole as well as nutation of the Earth axis. The revolution of the Earth around the Sun may not result in the change in its axis in the inertial space, and consequently does not affect the measurements of a star tracker.

#### Different coordinate systems in use

The coordinate systems used in the proposed method include the celestial coordinate system, ecliptic coordinate system, terrestrial coordinate system, and star tracker coordinate system.

**Figure 2:**
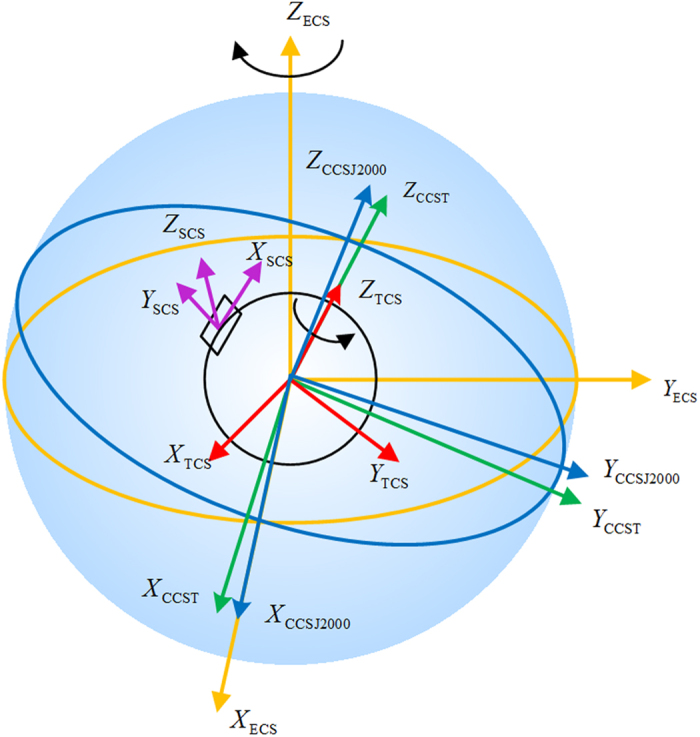
Definition of coordinate systems.

- (1) The Celestial Coordinate System is referred as CCS. In consideration of the effects of precession and nutation of the Earth, the CCS is time-related. To make system analysis convenient, a J2000.0 CCS is established internationally, expressed by CCSJ2000, as shown in Fig. 2. The J2000.0 CCS is established at 12 terrestrial dynamical time on January 1, 2000, with a *Z*-axis pointing towards the north pole of the Earth, an *X*-axis pointing towards the vernal equinox at the establishment time and *Y*-axis satisfying the right-hand rule with *X*, *Y*-axes. The Navigation stars in the original catalog star tracker are usually established based on the J2000.0 CCS and expressed in directional vectors and proper motion. Given the effect of precession, nutation, and so on, CCS rotates according to time. The Celestial Coordinate System at a certain time, expressed by CCST, can be acquired by eliminating the effect of precession and nutation based on the J2000.0 CCS.
- (2) The Ecliptic Coordinate System is referred as ECS. Its *X*_ECS_, *Y*_ECS_ and *Z*_ECS_ are shown in Fig. 2. The ECS is established at 12 terrestrial dynamical time on January 1, 2000, and remains constant. A revolution orbit of the Earth around the Sun is referred to as the ecliptic, and with the core of the Earth as a center, an X-axis pointing towards the vernal equinox at the establishing time and a Z-axis perpendicular to the ecliptic plane. The Y, X, and Z-axes of the ecliptic coordinate system satisfy the right-hand rule. The X-axis of the J2000.0 CCS coincides with the X-axis of the ECS. The angle between the Z-axis of the ECS and the Z-axis of the J2000.0 CCS is *ε*_0_. The celestial coordinate system revolves around the Z-axis of the ecliptic coordinate system every year by Δ*ψ*, which is referred to as the precession.
- (3) The Terrestrial Coordinate System is defined similar to that of the celestial coordinate system, except that with the Earth’s movement, the terrestrial coordinate system rotates around the Z-axis of CCS uniformly at an angular velocity Ω of 7.292115 × 10^−5^ rad/s. The terrestrial coordinate system is expressed by TCS as shown in Fig. 2.
- (4) The star tracker coordinate system is fixed with the star tracker and moves along therewith, with a center of the detector as a center of the star tracker coordinate system. The X and Y axes of the star tracker coordinate system are parallel to a row and a column of the detector, respectively. The Z, X, and Y axes of the star tracker coordinate system satisfy the right-hand rule. The star tracker coordinate system is expressed by SCS, for example, *X*_SCS_, *Y*_SCS_ and *Z*_SCS_ as shown in Fig. 2.

Given that navigation stars measured by the star tracker are all fixed stars and are far from the Earth, origins of the above mentioned four coordinate systems can be considered to be the same point. Hence, the transformation among the four coordinate systems includes only rotation transformation. The basic method of rotation transformation is as follows.

If *x*, *y*, *z* are coordinates in an original coordinate system, and (*x*′, *y*′, *z*′) are coordinates after the original coordinate system rotates, then


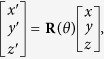


where coordinate transformation bases with respect to the *X*,*Y*, and *Z* axes are as follows:


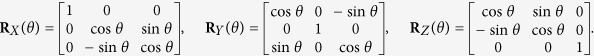


### Implementation of the AMSTA method

#### Transformation method of the attitude matrix

After installation, the current time *T* relative to J2000.0 is firstly input into the star tracker before it starts to work. From the vector, expressed by right ascension and declination (*α*, *δ*) of the navigation star, and proper motion parameters (*α*′, *δ*′), on the direction of right ascension and declination, the direction vector **v**_CCSJ2000_ of navigation star at time *T* in J2000.0 coordinate system can be determined by equation (1):


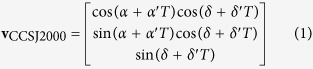


Along with the Earth’s motion, the star tracker can output the corresponding attitude information. The transformation procedure from output attitude quaternions to 3-axis accuracy is (the Implementation block diagram is in Supplementary Fig. S2):

- (1) Based on the directional vector in the star tracker system and the directional vector **v**_CCSJ2000_ in J2000.0 of the navigation star, optimal attitude quaternion **q**_*i*_ = [*q*_1_  *q*_2_  *q*_3_  *q*_4_] and relative shooting time *T* + Δ*t*_*i*_ can be obtained and exported.
- (2) From the quaternion **q**_*i*_, optimal matrix **A**_*q*_(*T* + Δ*t*_*i*_) can be deduced as equation (2):
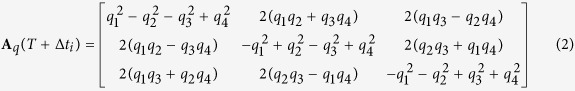
- (3) On the basis of the actual shooting time *T* + Δ*t*_*i*_, and the precession, nutation and rotation of the Earth, the accuracy measurement transformation matrix 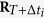 can be obtained by the following steps (a) to (d).

- (a) The transformation matrix **R**_ECS_(−*θ*_1_) is obtained from J2000.0 to ecliptic coordinate system. Based on the J2000.0 coordinate system (*X*_CCSJ2000_, *Y*_CCSJ2000_, *Z*_CCSJ2000_), the J2000.0 is rotated around the *X*-axis of J2000.0 counterclockwise by *ε*_0_ to obtain the ecliptic coordinate system (*X*_ECS_, *Y*_ECS_, *Z*_ECS_) as equation (3):
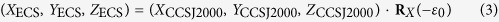
Therefore, **R**_ECS_(−*θ*_1_) = **R**_*X*_(−*ε*_0_), where **R**_*X*_ is the coordinate transformation basis.
- (b) The transformation matrix **R**_CCST_(−*θ*_2_) can be obtained from ECS (*X*_ECS_, *Y*_ECS_, *Z*_ECS_) to CCST (*X*_CCST_, *Y*_CCST_, *Z*_CCST_) at current time *T* through the following steps:

First, we can rotate the ecliptic coordinate system (*X*_ECS_, *Y*_ECS_, *Z*_ECS_) around the *Z*-axis clockwise by Δ*ψ* × *T*; Then, we can rotate the coordinate system obtained around the *X*-axis clockwise by *ε*_0_; The coordinate system obtained is rotated around the *X*-axis counterclockwise by *ε*_A_; The coordinate system obtained is rotated around the *Z*-axis clockwise by Δ*φ*; Finally, we rotate the coordinate system obtained about the *X*-axis clockwise by *ε*_A_ + Δ*ε*.

Thus, the celestial coordinate system (*X*_CCST_, *Y*_CCST_, *Z*_CCST_) at current time *T* containing precession and nutation terms is obtained. Δ*ϕ* and Δ*ε* represent a nutation in longitude and an oblique nutation, respectively. The transformation process of the celestial coordinate system (*X*_CCST_, *Y*_CCST_, *Z*_CCST_) can be expressed through the following equation (4):


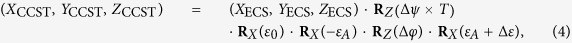


where **R**_*X*_ and **R**_*Z*_ are coordinate transformation bases. Therefore,


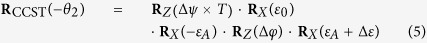


According to the IAU2000B nutation model^40^, *ε*_*A*_, the nutation in longitude (Δ*ϕ*) and the oblique nutation (Δ*ε*) in equation (5) can be determined by equation (6) respectively:


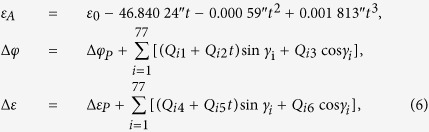


where Δ*φ*_*P*_ = −0.000 135″, Δ*ε*_*P*_ = 0.000 388″, *ε*_0_ = 84 381.448″, Δ*ψ* = 50.290″, *t* is the Julian century number starting from J2000.0 and is acquired based on current time (*T*).

In addition, in the above formulas, an argument *γ*_*i*_ is a linear combination of arguments which is determined as equation (7):


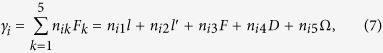


where *n*_*ik*_ is an integer, and *F*_*k*_ is a Delaunay argument related to positions of the Sun and the Moon. The expression for *F*_*k*_ is as equation (8):


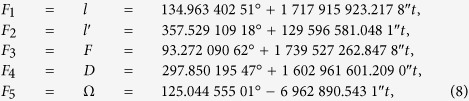


where *n*_*ik*_ and *Q*_*i*1_ − *Q*_*i*6_ in the nutation expression can be obtained from the website of the *International Earth Rotation and Reference Systems Service*^41^. Supplementary Tables S1 and Table S2 list the first 10 items of the coefficients.

- (c) The transformation matrix **R**_TCS_(−*θ*_3_) from CCST (*X*_CCST_, *Y*_CCST_, *Z*_CCST_) at time *T* to TCS (*X*_TCS_, *Y*_TCS_, *Z*_TCS_) at time *T* + Δ*t*_*i*_ can be deduced by rotating the CCST (*X*_CCST_, *Y*_CCST_, *Z*_CCST_) around the *Z*-axis of CCST counterclockwise at an angular velocity Ω = 7.292115 × 10^−5^ rad/s as equation (9).
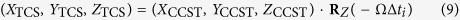
Therefore, **R**_TCS_(−*θ*_3_) = **R**_*Z*_(−ΩΔ*t*_*i*_).
- (d) The accuracy measurement conversion matrix 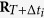 is obtained as equation (10):
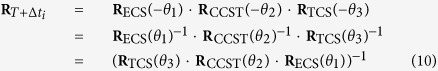
The steps (a–b) only need to be performed once, but the step (c) needs to be performed for each time or in real time to collect and convert data continuously. Thus, the accuracy measurement conversion matrix as varied with the actual shooting time (*T* + Δ*t*_*i*_) can be determined.
- (4) The accuracy measurement matrix can be obtained adopting the optimal attitude matrix **A**_*q*_(*T* + Δ*t*_*i*_) and accuracy measurement conversion matrix 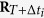:
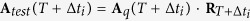
The directional vector **p**(*T* + Δ*t*_*i*_) of the three axes of the star tracker at the actual shooting time (*T* + Δ*t*_*i*_) can be determined from equation (11) on the basis of the accuracy measurement matrix **A**_*test*_(*T* + Δ*t*_*i*_).
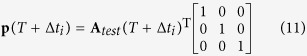

#### Evaluation of the star tracker accuracy

The angles (*α*_*i*_, *β*_*i*_, *ε*_*i*_), which are between the optimal vectors of three axes and vectors of *X*-axis, *Y*-axis and *Z*-axis at every actual shooting time (*T* + Δ*t*_*i*_) can be deduced based on the vectors of three axes **p**(*T* + Δ*t*_*i*_) as follows:

- (1) The three direction vectors of the star tracker **p**(*T* + Δ*t*_*i*_) are expressed by row vectors as equation (12):
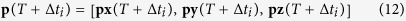
Each row vector is normalized.
- (2) The optimal vectors **p**_*opt*_(*T* + Δ*t*_*i*_) of *X*-axis, *Y*-axis and *Z*-axis of the star tracker can be acquired from above row vectors of three axes. The three row vectors [**px**_*opt*_(*T* + Δ*t*_*i*_), **py**_*opt*_(*T* + Δ*t*_*i*_), **pz**_*opt*_(*T* + Δ*t*_*i*_)] are calculated to have the minimum sums of the squares of the included angles with vectors [**px**(*T* + Δ*t*_*i*_), **py**(*T* + Δ*t*_*i*_), **pz**(*T* + Δ*t*_*i*_)] at shooting time (*T* + Δ*t*_*i*_) respectively. The optimal row vectors are normalized.
- (3) According to the optimal vectors **p**_*opt*_(*T* + Δ*t*_*i*_) of three axes and vectors of three axes **p**(*T* + Δ*t*_*i*_) at the actual shooting time (*T* + Δ*t*_*i*_), the cosine matrix **C** can be calculated using equation (13):
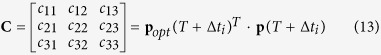
- (4) Based on the cosine matrix **C**, the angles (*α*_*i*_, *β*_*i*_, *ε*_*i*_), which are between the optimal vectors of three axes and vectors of *X*-axis, *Y*-axis and *Z*-axis at actual shooting time (*T* + Δ*t*_*i*_) can be obtained using equation (14):
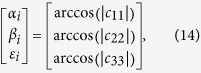

where (*α*_*i*_, *β*_*i*_, *ε*_*i*_) are within the range of 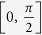.

Referring to the statistical regularities, the angles *η*_*i*_ between the optimal vector of one axis and other vectors of the same axis at different sampling time accord with the Gaussian distribution with mean of zero, and variance of *σ*^2^. However, given that angle measurement errors can only be positive, the probability density function is slightly different from the usual probability density function of Gaussian distribution, and can be represented as^42^ in equation (15):


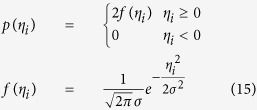


*σ* can be represented by the following equation (16):


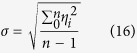


*α*_*i*_, *β*_*i*_, *ε*_*i*_ are expressed with *η*_*i*_ uniformly. *σ*_*α*_, *α*_*β*_, *σ*_*ε*_ can be obtained after substituting *α*_*i*_, *β*_*i*_, *ε*_*i*_ for *η*_*i*_ in above formula respectively. *n* is the sampling number.

**Figure 3:**
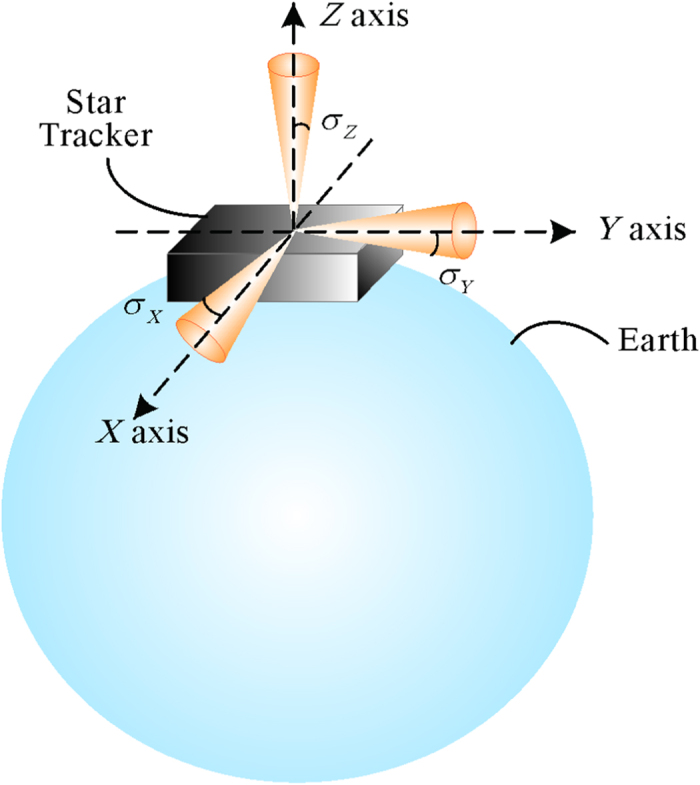
Pointing and rolling accuracy schematic of the star tracker.

The pointing and rolling accuracy schematic of the star tracker is shown in Fig. 3:

*α*_*i*_, *β*_*i*_, *ε*_*i*_ can reflect minor changes in the three axes brought about by error. This can be used as the evaluation criteria of the accuracy of the star tracker. The rolling accuracy of the star tracker can be expressed as 3*σ*_*α*_ (99.7%) or 3*σ*_*β*_ (99.7%), and the pointing accuracy can be expressed as 3*σ*_*ε*_ (99.7%).

The rolling accuracy has the same meaning with commonly-used ‘cross-boresight accuracy’.

### Real night sky experiments and the results

Real night sky experiment is an on-ground method of accuracy measurement that simulates as closely as possible the real performance of star trackers on orbit. The commonly used method is installing the star tracker on the telescope, and utilizing the rotation of the telescope to adjust the alignment of the star tracker to observe different region of the sky. Thus, the ability of the star tracker to identify different regions of the sky can be verified. Given that the telescope has a high rotational accuracy, the accuracy of the star tracker can be measured. However, in the research and development stage, it is usually inconvenient to use telescopes in addition to their high costs. Here we the proposed AMSTA method to test the star identification function and accuracy adopting the rotation of the Earth.

The rotational accuracy of the Earth is particularly high, and has few limitations on the measurement time and location. This property provides great facilities for the experiments. However, it is better to avoid the effect of factors, such as the moon and clouds, and therefore, it needs to choose the appropriate weather after comprehensive consideration. To observe the different regions of the sky, we can use a turntable or an adjustable tripod to adjust the pointing of the star tracker to accommodate various situations.

**Figure 4:**
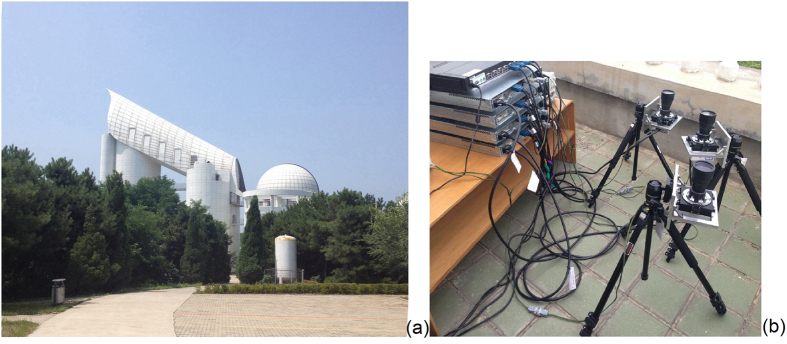
Experiment site at Xinglong observation station of National Astronomical Observatories of China(NAOC) (**a**) and the experiment devices (**b**).

The experimental conditions and experimental results are described and analyzed in the following portion. In the experiment, the exposure time is set to 192 ms. The experiment site is at the *Xinglong observation station of National Astronomical Observatories of China* (*NAOC*). The experiment ran from local time 20:00 on January 26^th^ to 5:00 on the 27^th^ in 2015. The optical axis is set to aim at the zenith. Figure 4 shows the experiment site at *NAOC*, and the experiment devices. The proposed accuracy measurement method can be performed for several star trackers in parallel operation.

**Figure 5:**
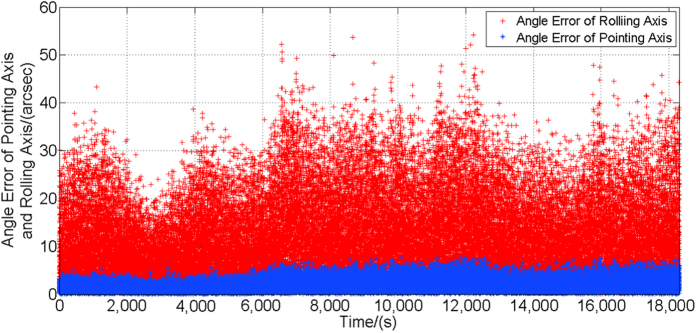
Angle error curves of pointing and rolling axes of the star tracker.

The AMSTA method uses the real sky observation method, and is based on the rotation of the Earth, which has the feature of high precision. Meanwhile, the method adopts the real stars as the image targets, and this is closer to the on-orbit situation. During the measurement, precession, nutation and rotation of the Earth have been processed. The following Fig. 5 is the angle error curve of pointing and rolling axis measured by the star tracker. The curves are results of around 18 000 s.

Statistical analysis shows that the pointing and rolling accuracy of the star tracker within the measurement time in the entire field of view is 3.30″ (3σ), and 23.96″ (3σ), respectively. The measurement results meet the accuracy requirements of the system specifications.


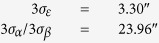


The proposed AMSTA method uses an explicit physical model. From the experimental data, the working performance in use such as changes of Euler angle, identified stars in the field of view can be confirmed as well (see Supplementary Figs S3–S5). Moonlight and clouds have little influence in the measurement process as shown in Supplementary Fig. S6 and Supplementary Video 1. Though the experiments are conducted at the observatory where the observation environment is good enough, the influence of atmosphere is inevitable. The effect of atmosphere on measurement accuracy is essentially related to the astronomical seeing. Better than 0.5″ of astronomical seeing is obtained at La Palma Observatory^43^. According to statistics, the astronomical seeing at Xinglong Observatory is around 1.1–1.5″^44^, and this can be ignored for 7″ star tracker. Higher accuracy star tracker needs higher environment conditions. As long as the choice of the environment is appropriate, the accuracy measurement method proposed in this manuscript is applicable for up to 1″ star tracker.

## Conclusions

The AMSTA method, which we proposed in this paper, utilizes the precision rotation of the Earth effectively. The star tracker is fixed on the ground, and the optic axis aims at the zenith. After corresponding transformations and processes of coordinate systems, the installation matrix relative to the terrestrial coordinate system can be obtained. Based on this matrix, changes in the three axes of a star tracker in the terrestrial coordinate system can be measured to obtain pointing and rolling accuracies. With verified experimental curves from real sky observations, the proposed method is proved to be effective and easy to implement and it can meet the accuracy requirements of a high-accuracy star tracker.

## Additional Information

**How to cite this article**: Sun, T. *et al.* An accuracy measurement method for star trackers based on direct astronomic observation. *Sci. Rep.* **6**, 22593; doi: 10.1038/srep22593 (2016).

## Supplementary Material

Supplementary Information

Supplementary Video 1
