# Supplementary material for: An accuracy measurement method for star trackers based on direct astronomic observation
Source: Sci Rep. 2016 Mar 7;6:22593. doi: 10.1038/srep22593 (PMC4984851; doi:10.1038/srep22593)
Supplement: Supplementary Information [file srep22593-s1.pdf]

An accuracy measurement method for star trackers based on direct astronomic observation

Ting Sun,[1,3] Fei Xing,[1,2,3*] Xiaochu Wang,[1,4] Zheng You,[1,3] and Daping Chu[2]

[1]*Department of Precision Instrument, Tsinghua University, Beijing, China*
[2]*Electrical Engineering Division, Department of Engineering, University of Cambridge, United Kingdom*
[3]*State Key Laboratory of Precision Measurement Technology and Instruments, Tsinghua University, Beijing, China*
[4]*Qian Xuesen Laboratory of Space Technology, China Academy of Space Technology, Beijing, China*
E-mail: xingfei@mail.tsinghua.edu.cn

Supplementary Figures, Tables and Video.

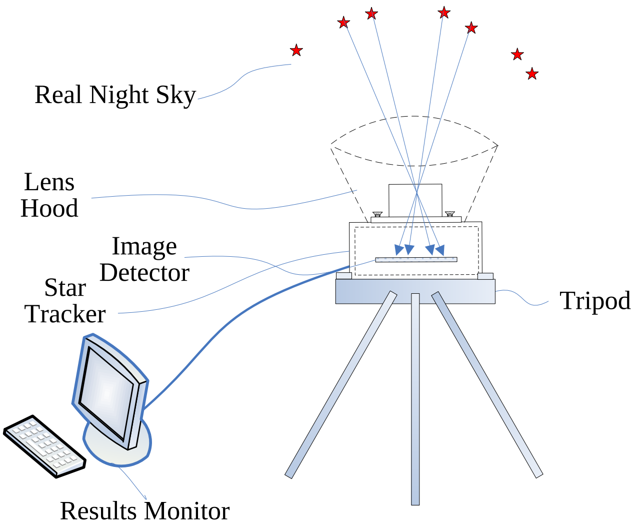

Supplementary Figure S1. Devices of the measurement system.

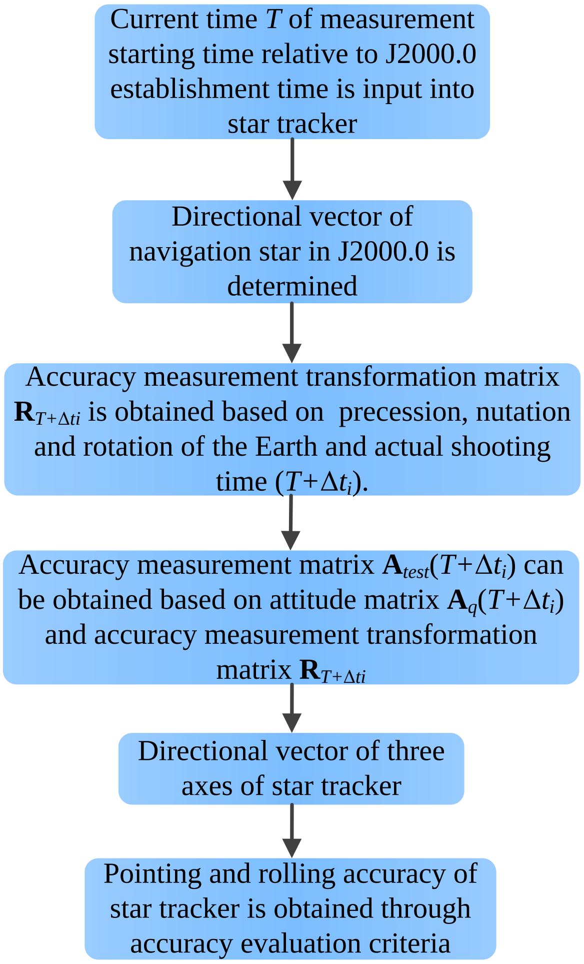

Supplementary Figure S2. Implementation block diagram of the proposed AMSTA method based on the transformation of the attitude matrix.

From the star pattern recognition results, the quaternion can be obtained by attitude estimation. Since the star tracker is fixed with the Earth, and rotates around *Z*-axis together with the Earth, the exported attitude matrix can represent the rotation result of the Earth relative to the inertial coordinate system. Supplementary Fig.S3 shows the Earth's rotation curve measured by the star tracker expressed by 3-2-1 Euler angles.

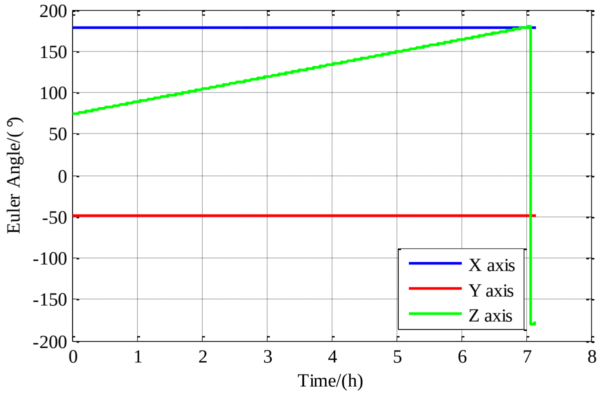

Supplementary Figure S3. Earth's rotation curve measured by the star tracker.

The measured angular velocity of the Earth's rotation around *Z*-axis is close to 15.04 °/h,

which is in good agreement with the theoretical angular velocity of the Earth's rotation. Euler angles around the other two axes (*X*-axis and *Y*-axis) remain unchanged, and the results are basically consistent with above theoretical analysis. Therefore, it can be verified that the star tracker can work well in the real night sky measurement.

To obtain the accuracy of the star tracker, the effect of the Earth's rotation, as well as the precession, nutation, and other factors have to be eliminated. In this case, Earth's rotation curves in the form of Euler angle measured by the star tracker are shown in Supplementary Fig.S4.

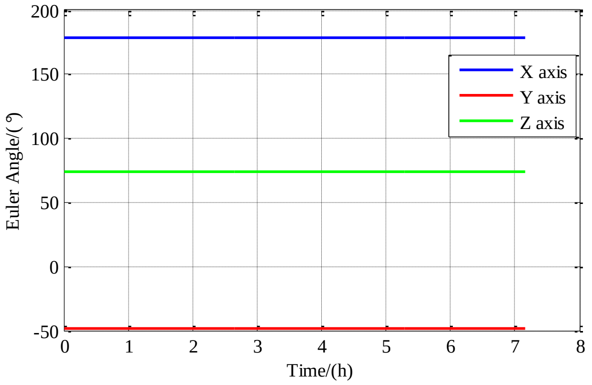

Supplementary Figure S4. Earth's rotation curves measured by the star tracker.

Supplementary Fig.S5 is the number of identified stars in the field of view.

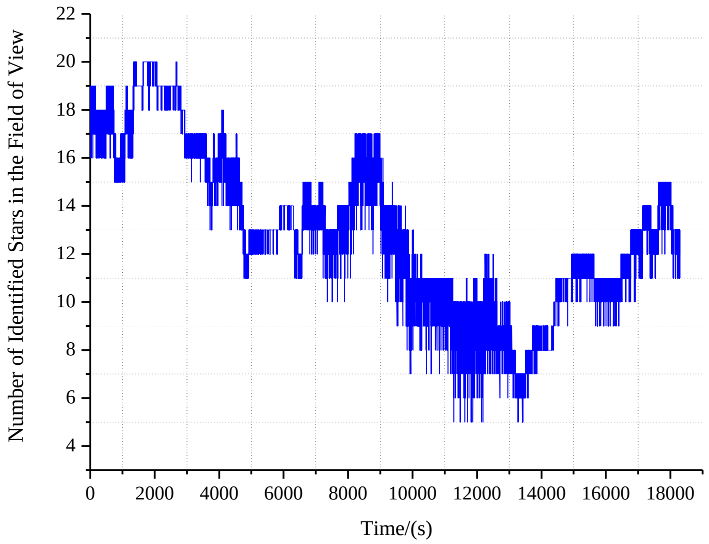

Supplementary Figure S5. Number of identified stars in the field of view.

Through the proposed method, the real performance in use can be proved as well. To verify the reliability of the system, the star tracker is measured under conditions of moonlit night and clouds while approaching dawn to test the work conditions with stray light. Supplementary Fig.S6 is the identification result when clouds exist. The red numbers represent the extracted number. Robustness and anti-interference of the star tracker can then be verified.

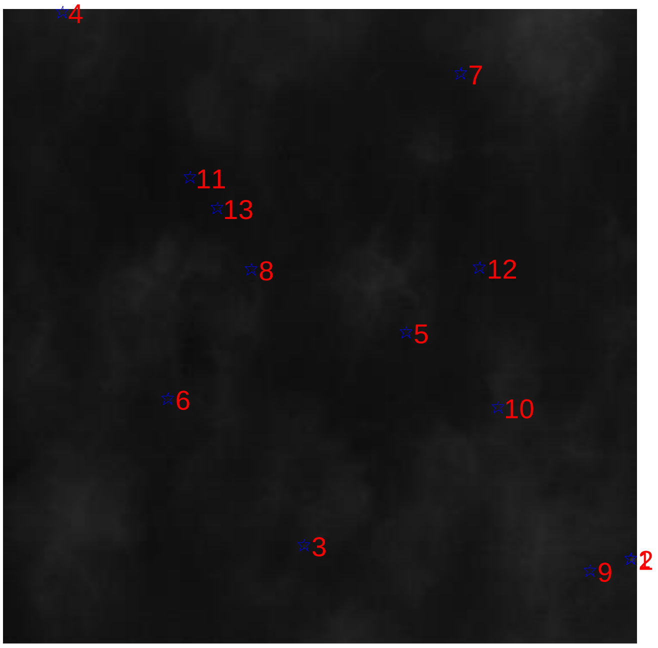

Supplementary Figure S6. Star tracker working under conditions of the clouds.

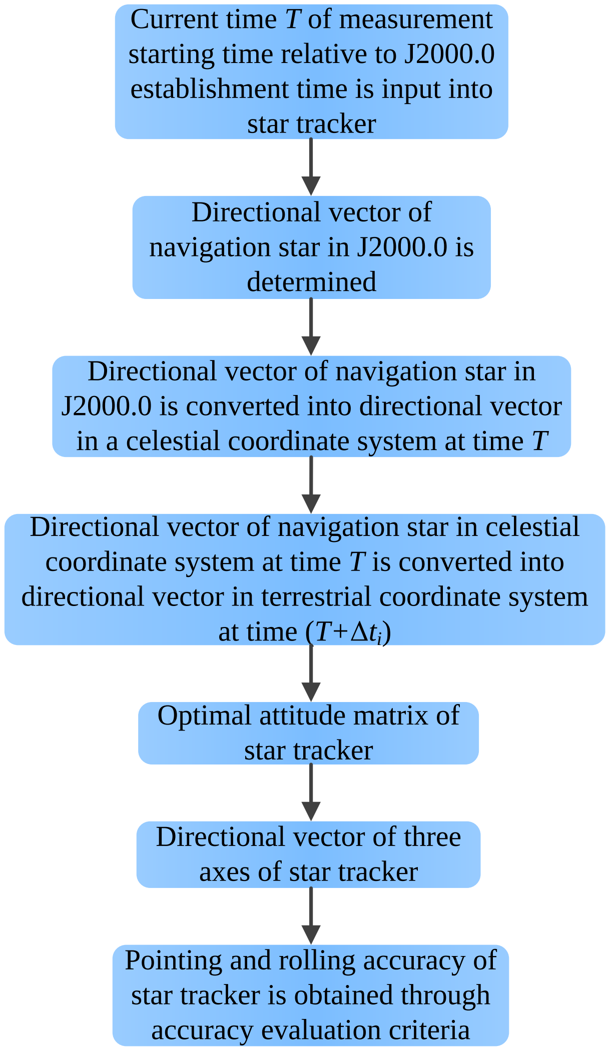

Supplementary Figure S7. Implementation block diagram of the proposed AMSTA method based on the transformation of the navigation star vector.

The following Supplementary Tables S1 and Supplementary Table S2 list the first 10 items of the coefficients.

Supplementary Table S1. Coefficients of anterior 10 angle of amplitude arguments in nutation expressions.

i	n_{i1}	n_{i2}	n_{i3}	n_{i4}	n_{i5}
1	0	0	0	0	1
2	0	0	2	-2	2
3	0	0	2	0	2
4	0	0	0	0	2
5	0	1	0	0	0
6	0	1	2	-2	2
7	1	0	0	0	0
8	0	0	2	0	1
9	1	0	2	0	2
10	0	-1	2	-2	2

Supplementary Table S2. Coefficients of anterior 10 arguments in nutation expressions.

i	Q_{i1}	Q_{i2}	Q_{i3}	Q_{i4}	Q_{i5}	Q_{i6}
1	-17206.4161	-17.4666	3.3386	9205.2331	0.9086	1.5377
2	-1317.0906	-0.1675	-1.3696	573.0336	-0.3015	-0.4587
3	-227.6413	-0.0234	0.2796	97.8459	-0.0485	0.1374
4	207.4554	0.0207	-0.0698	-89.7492	0.0470	-0.0291
5	147.5877	-0.3633	1.1817	7.3871	-0.0184	-0.1924
6	-51.6821	0.1226	-0.0524	22.4386	-0.0677	-0.0174
7	71.1159	0.0073	-0.0872	-0.6750	0.0000	0.0358
8	-38.7298	-0.0367	0.0380	20.0728	0.0018	0.0318
9	-30.1461	-0.0036	0.0816	12.9025	-0.0063	0.0367
10	21.5829	-0.0494	0.0111	-9.5929	0.0299	0.0132

Supplementary Video 1. Working performance of star tracker under conditions of the clouds.

Supplementary Method.

Transformation method of the navigation star vector

Along with the Earth's motion, the star tracker can output the corresponding attitude and star image information. The transformation procedure from output attitude quaternions to 3-axis accuracy is as shown in Supplementary Fig.S7:

(1) Current Navigation Star Vector Calibration: After installation, the current time T relative to J2000.0 is firstly input into the star tracker before it starts to work. From the vector, expressed by right ascension and declination (α,δ) of the navigation star, and proper motion parameters (α',δ'), on the direction of right ascension and declination, the direction vector $\mathbf{v}_{\mathrm{CCSJ2000}}$ of navigation star at time T in J2000.0 coordinate system can be determined by equation (1):

$$\mathbf{v}_{\mathrm{CCSJ2000}} = \begin{bmatrix} \cos(\alpha+\alpha'T)\cos(\delta+\delta'T) \\ \sin(\alpha+\alpha'T)\cos(\delta+\delta'T) \\ \sin(\delta+\delta'T) \end{bmatrix} \qquad (1)$$

(2) Precession Elimination: Depending on the relationship between CCSJ2000 and ECS, the vector of the navigation star at time T under ECS, denoted by $(\mathbf{v}_{\mathrm{ECS}})$, includes the counterclockwise rotation around X-axis of ECS by ε_0, and clockwise rotation around Z-axis of ECS by $\Delta\psi\times T$.

(3) Nutation Elimination: According to the IAU2000B nutation model, the vector $(\mathbf{v}_{\mathrm{ECS}})$ is rotated around the X-axis counterclockwise by ε_{A} .The obtained vector of the navigation star is then rotated around the Z-axis clockwise by $\Delta\varphi$. Finally, the resulting vector is rotated around the X-axis clockwise by $\varepsilon_{\mathrm{A}}+\Delta\varepsilon$, to obtain the directional vector $(\mathbf{v}_{\mathrm{CCST}})$ of the navigation star in the CCS at current time (T). This vector contains nutation terms, in which the $\Delta\phi$ and $\Delta\varepsilon$ represent a nutation in longitude and an oblique nutation, respectively.

The whole process can be expressed by equation (2):

$$\begin{aligned} \mathbf{v}_{\mathrm{CCST}} =& \mathbf{R}_X(-(\varepsilon_A+\Delta\varepsilon))\cdot\mathbf{R}_Z(-\Delta\varphi)\cdot\mathbf{R}_X(\varepsilon_A)\cdot \\ & \mathbf{R}_X(-\varepsilon_0)\cdot\mathbf{R}_Z(-\Delta\psi\times T)\cdot \\ & \mathbf{R}_X(\varepsilon_0)\ \mathbf{v}_{\mathrm{CCSJ2000}}, \end{aligned} \qquad (2)$$

where $\mathbf{R}_X$ and $\mathbf{R}_Z$ are coordinate transformation bases.

ε_A, the nutation in longitude ($\Delta\phi$) and the oblique nutation ($\Delta\varepsilon$) are determined by equation (3), respectively:

$$\varepsilon_A=\varepsilon_0-46.840\ 24''t-0.000\ 59''t^2+0.001\ 813''t^3,$$

$$\Delta\varphi = \Delta\varphi_P + \sum_{i=1}^{77}[(Q_{i1} + Q_{i2}t)\sin\gamma_{\mathrm{i}} + Q_{i3}\cos\gamma_i],$$

$$\Delta\varepsilon = \Delta\varepsilon_P + \sum_{i=1}^{77}[(Q_{i4} + Q_{i5}t)\sin\gamma_i + Q_{i6}\cos\gamma_i], \quad (3)$$

where $\Delta\varphi_P = -0.000\ 135''$, $\Delta\varepsilon_p = 0.000\ 388''$, $\varepsilon_0 = 84\ 381.448''$, $\Delta\psi = 50.290''$. t is the Julian century number starting from J2000.0 and is deduced based on the current time (T).

In addition, in the above formulas, an argument γ_i is a linear combination of arguments which is obtained as equation (4):

$$\gamma_i = \sum_{k=1}^{5} n_{ik}F_k = n_{i1}l + n_{i2}l' + n_{i3}F + n_{i4}D + n_{i5}\Omega, \quad (4)$$

where n_{ik} is an integer, and F_k is a Delaunay argument related to positions of the Sun and the Moon. The expression for F_k is as equation (5):

$$F_1 = l = 134.963\ 402\ 51° + 1\ 717\ 915\ 923.217\ 8''t,$$

$$F_2 = l' = 357.529\ 109\ 18° + 129\ 596\ 581.048\ 1''t,$$

$$F_3 = F = 93.272\ 090\ 62° + 1\ 739\ 527\ 262.847\ 8''t,$$

$$F_4 = D = 297.850\ 195\ 47° + 1\ 602\ 961\ 601.209\ 0''t,$$

$$F_5 = \Omega = 125.044\ 555\ 01° - 6\ 962\ 890.543\ 1''t \quad (5)$$

(4) Directional vector along the three axes of the star tracker can be acquired based on the directional vector ($\mathbf{v}_{\mathrm{CCST}}$) by the following steps (a) to (c).

(a) The directional vector ($\mathbf{v}_{\mathrm{CCST}}$) in CCS at current time (T) is converted into the directional vector ($\mathbf{v}_{\mathrm{TCS}}$) in the TCS at the actual shooting time ($T + \Delta t_i$) around Z-axis of the celestial coordinate system counterclockwise at an angular velocity $\mathbf{\Omega}$ =7.292115 $\times 10^{-5}$ rad/s as shown in equation (6):

$$\mathbf{v}_{\mathrm{TCS}} = \mathbf{R}_{\mathrm{Z}}(\Omega\Delta t_i)\mathbf{v}_{\mathrm{CCST}} \quad (6)$$

This step needs to be conducted at each time or in real time to collect and convert data continuously. Therefore, coordinate data of the navigation star in TCS varied with the actual shooting time $T + \Delta t_i$ can be obtained.

(b) According to the directional vector ($\mathbf{v}_{\mathrm{TCS}}$) in TCS, the optimal attitude matrix $A_q(T + \Delta t)$ is obtained by minimizing the following objective function $J\left(A_q\left(T + \Delta t\right)\right)$ through QUEST method as shown in equation (7):

$$J\left(\mathbf{A}_q\left(T + \Delta t_i\right)\right) = \frac{1}{2}\sum_{i=1}^{n}\lambda_i\left\|\mathbf{w}_i - \mathbf{A}_q\left(T + \Delta t_i\right)v_i\right\|^2, \quad (7)$$

where $\mathbf{w}_i, \mathbf{v}_i$ represent a directional vector of the navigation star in a star tracker coordinate system and a directional vector of the navigation star in the terrestrial coordinate system, respectively; λ_i represents a weighing coefficient; and $\sum\lambda_i = 1$.

(c) Based on the optimal attitude matrix $A_q(T + \Delta t)$ of the star tracker, the vector $\mathbf{p}(T + \Delta t_i)$ of three axes of the star tracker can be calculated from equation (8) at the actual shooting time ($T + \Delta t_i$).

$$\mathbf{p}(T+\Delta t_i)=\mathbf{A}_q(T+\Delta t_i)^{\mathrm{T}}\begin{bmatrix}1 & 0 & 0\\ 0 & 1 & 0\\ 0 & 0 & 1\end{bmatrix} \tag{8}$$
